# A case report of cognitive behavioural and emotional therapy for depression in an ultra-high risk patient

**DOI:** 10.1192/j.eurpsy.2023.1033

**Published:** 2023-07-19

**Authors:** R. Ben Massoued, A. Aissa, A. Larnaout, R. Lansari, U. Ouali, R. Jomli

**Affiliations:** 1 Psychiatry Departement D; 2 Psychiatry Departement A, Razi Hospital, Manouba, Tunisia

## Abstract

**Introduction:**

Psychotic disorders are associated with a degree of disability that is much more considerable if the duration of untreated psychosis is prolonged. This fact highlights the importance of early intervention strategies among individuals at ultra-high risk (UHR) for psychosis, often struggle with depressive symptoms.

**Objectives:**

The objective of this work was to evaluate the effectiveness of cognitive-behavioral and emotional therapy on depressive symptoms in a patient at high risk of psychosis.

**Methods:**

This is a detailed case report of a young adult at UHR for psychosis who was referred to psychiatry department “A” at Razi Hospital for treatment of depression symptoms.

The patient had benefited from 15 sessions of cognitive-behavioral and emotional psychotherapy, over eight months, from July 2021 to February 2022, at the rate of one 45-minute session per two weeks. The main psychotherapeutic techniques used were: behavioral activation, cognitive restructuring and relaxation.

An initial and final assessment was performed by the Hamilton Depression Scale and by the comprehensive assessment of mental states at risk.

**Results:**

The clinical case illustrated concerns a 21-year-old female with a state of high risk of psychosis, suffering from depression symptoms that had been worsen since two years.

As the therapy progressed, an improvement in depressive symptoms and functioning has been noticed, by a decrease in the score of the Hamilton scale, from 28 to 11, with a response estimated at 61% and a score for social functioning and professional, initially estimated between 21 and 30, to became between 41 and 50 after therapy.

The active participation of the patient, and her assiduity were important factors in this success.

**Conclusions:**

Cognitive-behavioral and emotional therapies for depressive symptoms could constitute an effective intervention approach for subjects at high risk of psychosis, allowing the improvement of the prognosis of psychotic disorders.

**Disclosure of Interest:**

None Declared

